# The Cellular Composition of Bovine Coccygeal Intervertebral Discs: A Comprehensive Single-Cell RNAseq Analysis

**DOI:** 10.3390/ijms22094917

**Published:** 2021-05-06

**Authors:** Martina Calió, Benjamin Gantenbein, Marcel Egli, Lucy Poveda, Fabian Ille

**Affiliations:** 1 Tissue Engineering for Orthopaedics & Mechanobiology (TOM), Department for BioMedical Research (DBMR), University of Bern, 3008 Bern, Switzerland; martina.calio@students.unibe.ch (M.C.); benjamin.gantenbein@dbmr.unibe.ch (B.G.); 2 Department of Orthopaedic Surgery and Traumatology, Inselspital, Bern University Hospital, 3010 Bern, Switzerland; 3 Space Biology Group, Institute of Medical Engineering, School of Engineering and Architecture, Lucerne University of Applied Sciences and Arts, 6052 Hergiswil, Switzerland; marcel.egli@hslu.ch; 4 Functional Genomics Center Zurich, Swiss Federal Institute of Technology, University of Zurich, 8057 Zurich, Switzerland; lucy.poveda@fgcz.uzh.ch

**Keywords:** intervertebral disc, nucleus pulposus, annulus fibrosus, RNA sequencing, bovine

## Abstract

Intervertebral disc (IVD) degeneration and its medical consequences is still one of the leading causes of morbidity worldwide. To support potential regenerative treatments for degenerated IVDs, we sought to deconvolute the cell composition of the nucleus pulposus (NP) and the annulus fibrosus (AF) of bovine intervertebral discs. Bovine calf tails have been extensively used in intervertebral disc research as a readily available source of NP and AF material from healthy and young IVDs. We used single-cell RNA sequencing (scRNAseq) coupled to bulk RNA sequencing (RNAseq) to unravel the cell populations in these two structures and analyze developmental changes across the rostrocaudal axis. By integrating the scRNAseq data with the bulk RNAseq data to stabilize the clustering results of our study, we identified 27 NP structure/tissue specific genes and 24 AF structure/tissue specific genes. From our scRNAseq results, we could deconvolute the heterogeneous cell populations in both the NP and the AF. In the NP, we detected a notochordal-like cell cluster and a progenitor stem cell cluster. In the AF, we detected a stem cell-like cluster, a cluster with a predominantly fibroblast-like phenotype and a potential endothelial progenitor cluster. Taken together, our results illustrate the cell phenotypic complexity of the AF and NP in the young bovine IVDs.

## 1. Introduction

Intervertebral disc (IVD) degeneration is a complex disease for which resolutive therapies are not yet available. Attempts to regenerate the tissue are complicated by the lack of knowledge on the exact cellular composition of the disc. Many studies have tracked and proven the developmental characteristics of IVDs [1,2,3]. However, little is known about the type of cells populating the mature tissue. IVDs are cushion-like structure between the vertebrae that act as stress and impact absorbers and as a protection for the spinal nerves. The IVD poses extreme conditions for cells to live in, leading to a hyperosmotic, avascular and acidic environment [4]. They provide viscoelastic and mechanical properties with shock-absorbing capacity that allow bending, twisting and loading of the upper body [5,6,7].

Structurally, the IVD is composed of three main compartments: the cartilaginous endplate (CEP), the annulus fibrosus (AF) and the nucleus pulposus (NP) [8]. The CEP, a thin layer of hyaline cartilage at the interface with the vertebrae, contains chondrocytes embedded in an aggrecan and collagen type II rich extracellular matrix [8,9]. It is composed of a network of collagen fibrils and hydrated proteoglycan molecules. Vascularized by microscopic vessels during development, they disappear once the IVD has reached maturity [9]. The annulus fibrosus (AF) is a compartment originating from mature somites. It is believed that the AF develops from the mesenchymal cells of the sclerotome that generate the connective tissues of the future axial skeleton [10]. The annulus is connected to the CEP via its lamellae, structures made of fibrous cartilage and mainly composed of water and concentric sheets of collagen fibers [5]. The lamellae, oriented at various angles, enclose the nucleus pulposus (NP).

The NP, which is believed to have derived from the notochord [11,12,13], is composed of a gel-like material with a loose network of collagen fibers [1,6,14,15,16]. On a cellular level, the outer human AF is populated by elongated and fusiform cells that extend in the long axis of the fibrils. The cells of the inner AF, instead, are spherical in shape, and many resemble chondrocytes [3]. The annulus fibrosus is believed to retain the capacity to regenerate itself during the neonatal phase, and this is then lost during maturation [17]. Annulus fibrosus cell (APC) markers described in the literature include *Col1a1, Col4, Lam1* and *Thy1*, among others [10,18,19,20,21,22,23,24]. Instead, the young NP presents an abundant extracellular matrix (ECM) that separates NP cells (NPC) [8]. Numerous NPC type populations have been described in the literature. Among them, there are large cells with numerous vacuoles that are believed to be notochordal remnants [25]. NPC progenitors have also been described in the healthy IVD that are TIE2+ and GD2+ positive [26]. Additionally, cells that express a mesenchymal stem cell phenotype have also been reported [27]. The NPC population changes with aging and degeneration leading to the disappearance of the aforementioned progenitor cells and vacuoled cells [27,28,29,30]. There are a wide variety of NPC biomarkers such as *Krt8, Krt18, Krt19, Ca12, ACAN, Col1a2* and *Col2a1*, described in the literature [19,31,32,33,34,35]. Additional, notochordal markers such as Brachyury, *FOXA2, CHRD, NOG* and *LGALS3* are also often used to track NP cells lineage [36,37,38,39,40], together with *NCAM1*, a2 macroglobulin and desmocollin 2 [36,41,42,43]. The mature IVD presents a very limited regenerative capacity [44]. However, a comprehensive profile of all of the NPC in this compartment is still missing [2,20,45]. This, in turn, hampers any attempts of regenerative therapies in patients suffering from disc degeneration which is still one of the leading causes of disability worldwide [2,46,47].

The purpose of our study was to deconvolute the cell composition in the AF and NP of healthy bovine IVD. Bovine IVDs are a widely used animal model for spine research as they offer the possibility to examine young and healthy tissues. Furthermore, the bovine model, while not a prefect substitute for the human model, has shown remarkable similarities in tissue specific marker expressions to the human model [41,48]. To reach our goal, we performed 10× Genomics single-cell RNA sequencing together with bulk RNAseq. Single-cell RNA sequencing is a powerful and innovative technique, which allowed the individual transcriptome analysis of 10 k NPCs and 10 k AFCs from two bovine tails.

## 2. Results

### 2.1. Single-Cell RNAseq Experimental Design

Eight samples were processed for whole transcriptome analysis using single-cell RNAseq sequencing (Table 1). Four samples were collected per biological replicate, i.e., the nucleus pulposus and annulus fibrosus from both, proximal and distal intervertebral discs of two bovine tails. Due to the nature of the composition of IVDs entailing a low cell number, each NP or AF sample consisted of a pool of three adjacent IVDs from either the distal or proximal region. To obtain a cell suspension, the ECM was digested before further processing.

### 2.2. Bulk RNAseq Experimental Design

High-throughput transcriptome analysis, also referred to as bulk RNAseq, has become a standard tool for identifying differentially expressed genes between two or more sample groups. scRNAseq is a lot noisier due to technical and biological influences, compared to bulk RNAseq [49,50]. Three biological replicates (three bovine tails) were used to isolate cells for the bulk RNAseq analysis. Similar to the scRNAseq samples, distal and proximal NP and AF samples were processed for each of the three biological replicates. Tissue from two IVDs was pooled for each sample (Table 2), and the ECM was digested overnight to obtain a cell suspension. The cell suspension was then subjected to bulk RNAseq.

### 2.3. Cell Clustering Using Single-Cell RNAseq Data

**Figure 1:**
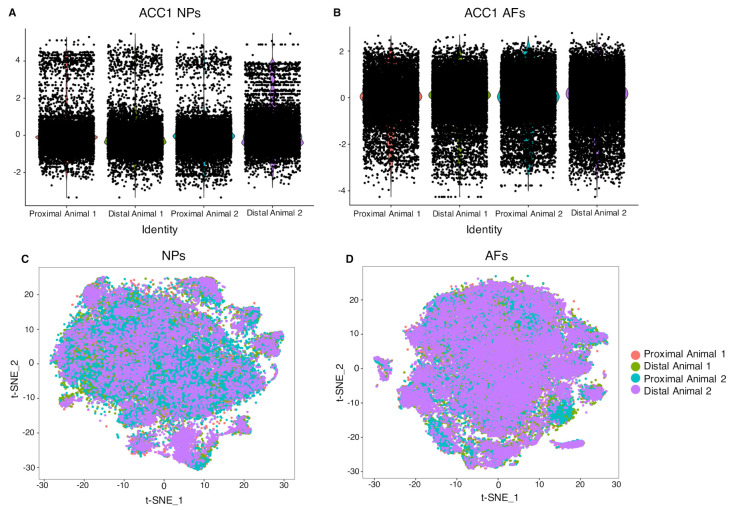
Canonical correlation analysis (CCA). CCA identifies common sources of variation between the datasets for NPs (**A**) and AFs (**B**). The CCA aligned subspaces ACC1 and ACC2 for each condition are plotted as violin plots with the points reflecting the data distribution. The plots reveal high correlation between proximal and distal NPs and AFs within and between animals. t-SNE plot of four CCA corrected NPs samples (**C**) and four CCA corrected AFs samples (**D**). Each dot represents a cell and the different colors are assigned to different samples. The location of a cell is determined by its gene expression profiles, therefore cells that localized in close proximity exhibit a high correlation in their gene expression pattern. In both graphs, the four samples contribute in a comparable way in populating each region of the graph, showing a good overlap among all the NPs (**A**) and among all the AFs (**B**).

To comprehensively characterize subpopulations of NPs and AFs in either distal or proximal discs, we used the data from the scRNAseq experiments. Cell populations within each of the samples can be clustered according to their single-cell transcriptome profile expression values utilizing graph-based cluster method to acquire unsupervised cell clustering results. To obtain an impression of the overall gene expression variance within the NPs and the AFs, we performed a canonical correlation analysis (CCA) and corrected the datasets by aligning the relevant canonical correlations (CCs). The aligned CCs (ACC) for the NPs (Figure 1A) and the AFs (Figure 1B) exhibit a high correlation between the samples and animals. To better visualize the correlation, we performed t-stochastic neighbor embedding (t-SNE). The overlap of the samples in each t-SNE plot showed a high expression profile correlation among the NP cell populations (Figure 1C) and among the AF cell populations (Figure 1D), within and between animals, respectively.

**Figure 2:**
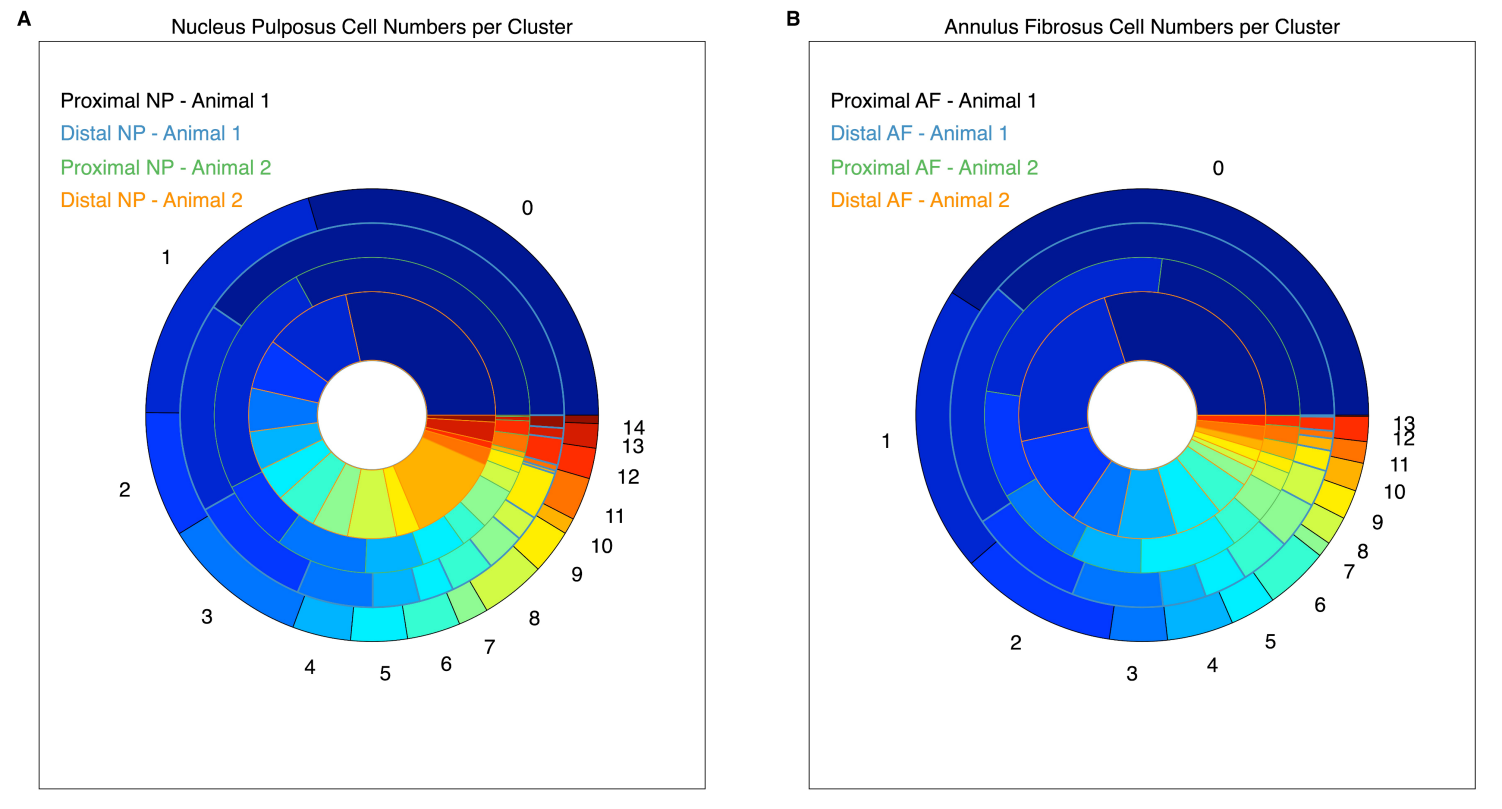
Cell cluster size correlation. The graphical alignment of cell cluster sizes within the NPs (**A**) and the AFs (**B**) shows a high correlation (r > 0.85) within the datasets. The numbers placed around the piechart are cell cluster numbers, with the largest cluster starting at number zero. Each ring represents one sample and each color within the ring represents one cluster.

With the apparent expression profile proximity in the t-SNE plot, we performed a cell cluster analysis and used cell cluster size correlation as a metric to better understand our sample datasets. A size comparison of the cell clusters in the NP samples (Figure 2A) and the AF samples (Figure 2B) served as metric. The graphical representation in Figure 2 shows a high correlation between cell cluster sizes of the NP samples and the AF samples. The correlation coefficients for the NP samples are higher than 0.85 and for the AF samples higher than 0.87. A correlation matrix with hierarchical clustering (Figure S1) shows that the differences of the distal and proximal NPs are higher than the differences between the animals. Conversely, the differences of the AFs between the animals are larger than between the proximal and distal AFs. This may indicate possible temporal shifts in development between NPs and AFs.

**Figure 3:**
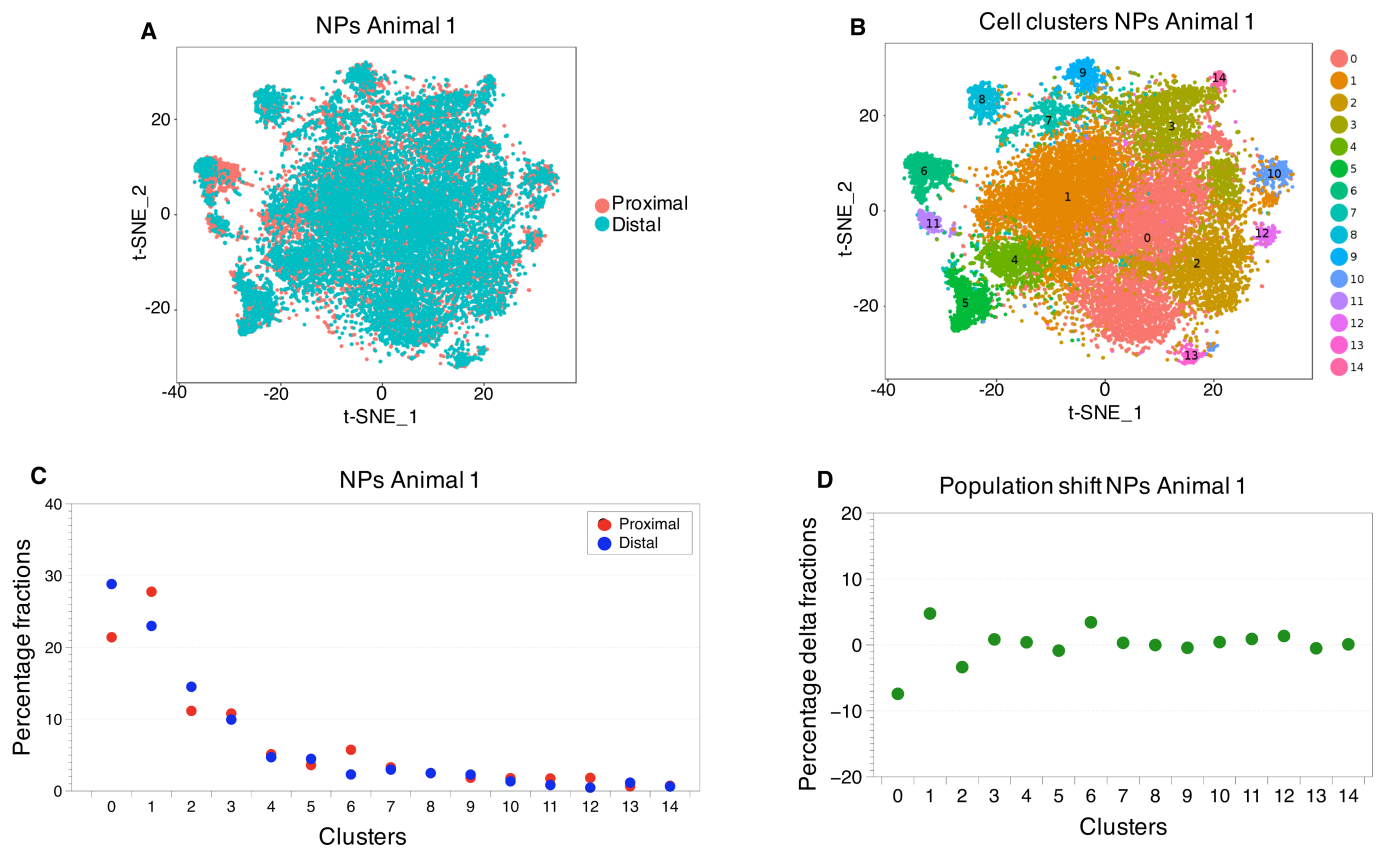
Cell cluster distributions between proximal and distal NPs.The first t-SNE plot (**A**) provides a visual measure for similarity for the proximal and distal NPs. The graphical overlap stands for the amount of similarity in gene expression profiles of the distal and proximal NPs. The second t-SNE plot (**B**) shows the identified cell clusters. (**C**) The population sized within a cell cluster. The first dot plot (**C**) is a representation of the percentage of the fractions of cells per cluster, normalized to the total amount of cells in each corresponding sample (see Table S1 for the original data). The population shift observed between the proximal and the distal samples, is described in terms of number of cells that compose a cluster in each sample. (**D**) The difference in percentage between the cell cluster sizes of the proximal and distal NPs, indicating the amount of correlation between the samples. (Animal 2, in Figure S2 and Table S3).

Using unsupervised clustering, the NP cell populations (Figure 3A) exhibited 15 distinct cell clusters (Figure 3B). This analysis revealed that proximal and distal NPs identified mostly overlapping cell populations (Figure 3A) resulting from a high correlation of expression profiles and some shifts in the percentage fractions of some of the most abundant cell populations (Figure 3C).

**Figure 4:**
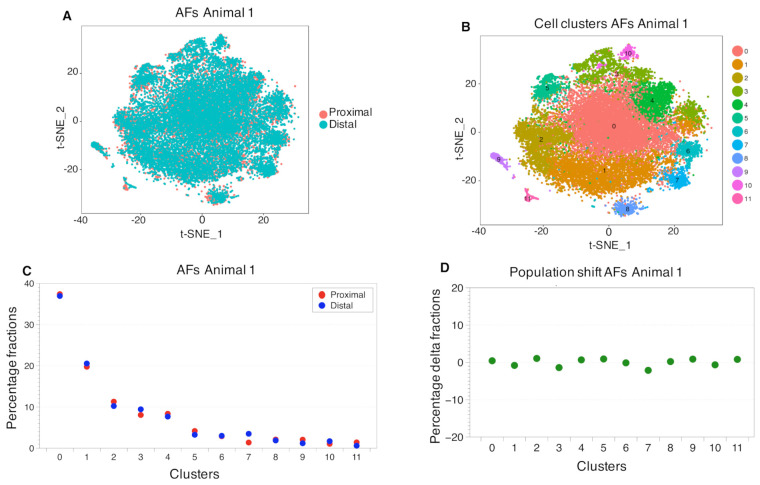
Cell cluster distributions between proximal and distal AFs. The first t-SNE plot (**A**) provides a visual measure for similarity for the proximal and distal AFs. The graphical overlap stands for the amount of similarity in gene expression profiles of the distal and proximal AFs. The second t-SNE plot (**B**) shows the identified cell clusters. (**C**) The population sized within a cell cluster. The first dot plot (**C**) is a representation of the percentage of the fractions of cells per cluster, normalized to the total amount of cells in each corresponding sample (see Table S2 for the original data). The population shift observed between the proximal and the distal samples, is described in terms of number of cells that compose a cluster in each sample. (**D**) The difference in percentage between the cell cluster sizes of the proximal and distal NPs, possibly indicating developmental transitions in cells. (Animal 2, in Figure S3 and Table S4).

In regards to the AF samples, the overlap between distal and proximal cell cluster populations was even higher (Figure 4A), with 12 main cell clusters identified (Figure 4B) and no apparent shift of cell populations (Figure 4C).

### 2.4. Differential Gene Expression Analysis from Bulk RNAseq

**Figure 5:**
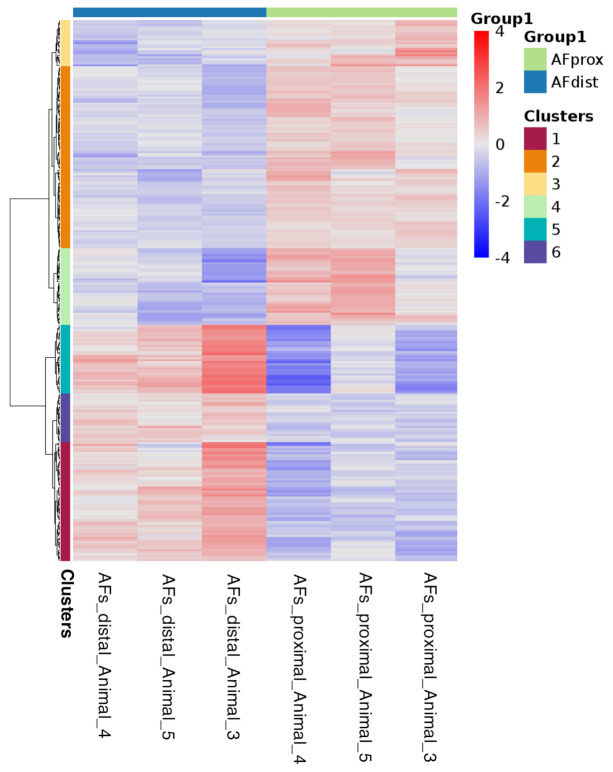
Heat map bulk AFs. Heat map for the differential genes expression for proximal and distal AFs (bulk RNAseq).

To further investigate potential phenotypic differences across all samples, we performed differential expression analysis on bulk RNAseq data. This analysis revealed that within the groups, i.e., NPs and AFs, samples are highly correlated and clustered together (Figures S4 and S5) as with the scRNAseq data. Firstly, we investigated other differences along the rostrocaudal axis within the groups, i.e., differential gene expression analysis from the proximal and distal NPs as well as AFs. Hierarchical clustering was applied to the data and plotted as a heat map. There were no significant differences in the proximal and distal NP expression profiles (data not shown). However, the AFs provided distinct expression patterns for the proximal and the distal AFs after gene clustering (Figure 5). Gene Clusters 1, 5 and 6 are upregulated in the distal AFs when compared to the proximal AFs. However, the opposite seems true for the gene Clusters 2–4, which are instead downregulated in the distal AFs.

**Figure 6:**
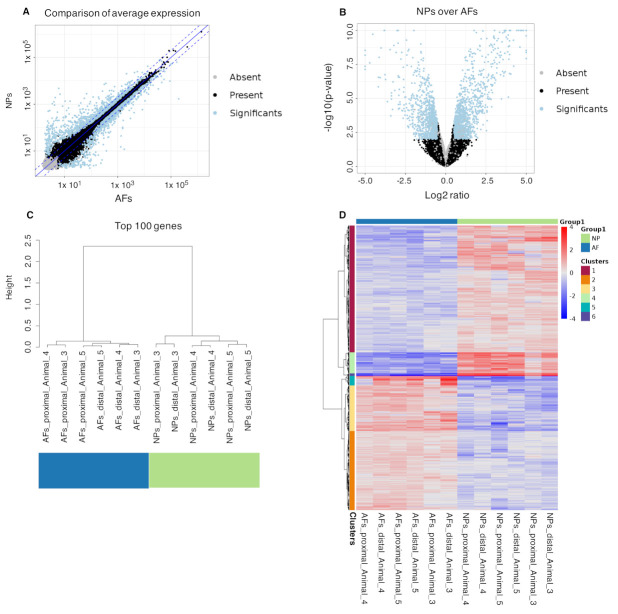
Comparison between NPs and AFs in the bulk RNAseq data. In the correlation graphic (**A**) is present a light correlation in the gene expression between all the (6) NPs and (6) AFs, with relevant differences. The volcano plot (**B**) shows a high number of significant differentially expressed genes with relevant fold changes between NPs and AFs. The dendrogram (**C**) compares the top 100 most expressed genes in the NPs and AFs. This shows a clear distinction between the gene expression of the NPs and the AFs, that clustered separately. Intra group, the NPs samples cluster per animal. In the AFs, instead, Animal 5 clustered together, while, for the Animals 3 and 4, the clustering occurs between the two proximal and the two distal samples. The heat map (**D**) shows a very clear distinction in the gene expression pattern of the NPs over the AFs. Here, the gene Clusters 1, 4 and 6 are upregulated in the NPs, while the gene Clusters 2, 3 and 5 are downregulated.

In a further analysis, we looked at the significant differential gene expression across all AFs and NPs. A hierarchical clustering was performed on the top 100 expressed genes of all samples and the data was normalized over all datasets. Figure 6A shows a good correlation in expression levels between the AF and NP groups. Figure 6B depicts the statistical significance of each gene against the fold change in expression level (Log2FoldChange), whereas Figure 6C shows the expressed genes that are up- or downregulated in either group and are statistically significant, which hints at biologically meaningful tissue profiles. Hierarchical clustering of the differentially expressed genes showed distinct gene clusters in both groups, AFs and NPs (Figure 6D).

### 2.5. Integration of the Single-Cell RNAseq Data with the Bulk RNAseq Data

**Figure 7:**
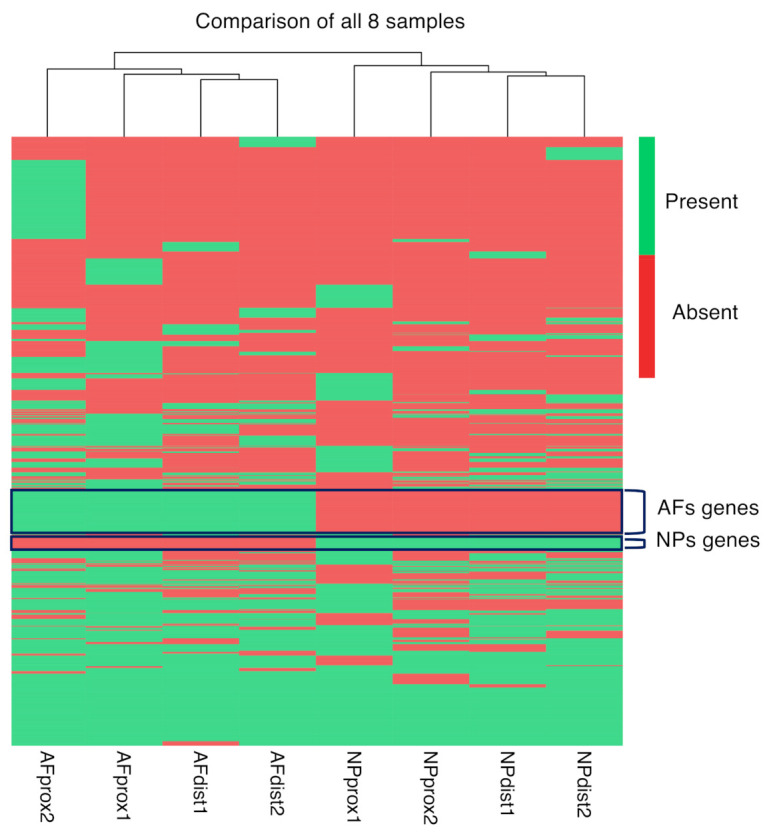
Heat map scRNAseq. Heat map depicting the presence and absence of expressed genes (top cluster markers) in the proximal and distal AFs and NPs of Animals 1 and 2, scRNAseq data. The gene clusters that are expressed exclusively in AFs and NPs are of particular interest as potential markers.

We went back to our scRNAseq data to identify genes that are exclusively expressed in either the NP or the AF samples. The heat map in Figure 7 depicts two sets of genes that are uniquely expressed in either all NPs or all AFs.

**Figure 8:**
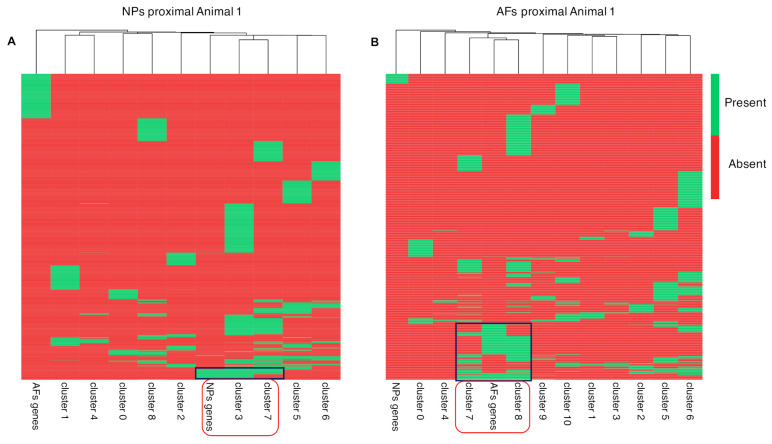
Heat map scRNAseq cluster. Heat map of exclusive AF and NP genes in the scRNAseq data, expressed in the NPs proximal and and AFs proximal of Animal 1. (**A**) The first heat map depicts the cell clusters that express NP genes. (**B**) The second heat map depicts cell clusters that express AF genes. The dendrogram at the top is a function of the amount of commonly expressed genes.

**Figure 9:**
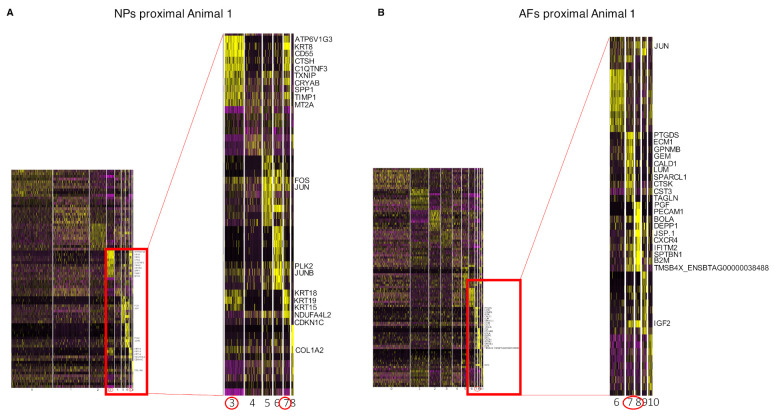
Genes that are highly expressed in scRNAseq clusters. Genes that are highly expressed in the clusters of interest in the scRNAseq data. (**A**) From the NPs proximal Animal 1, the Clusters 3 and 7 previously identified were selected. (**B**) From the AFs proximal Animal 1, the Clusters 7 and 8 previously identified were selected. The focus was pointed on the genes that are highly expressed in the clusters of interest. Higher expression value are highlighted in yellow.

The two extracted gene clusters were subsequently mapped to cell clusters to identify the cell populations within the AF and NP that express those exclusive genes (Figure 8). In both sample sets, the AFs and NPs, we identified two cell populations that expressed the previously identified unique expressed genes. In the NPs, cell population/Clusters 3 and 7 showed the highest overlap with the uniquely identified NP genes (Figure 8A), whereas cell population/Clusters 7 and 8 had the highest overlap in the AF unique genes (Figure 8B). Subsequently, we investigated the expression levels of the unique set of AF and NP genes in an expression heat map (Figure 9).

**Figure 10:**
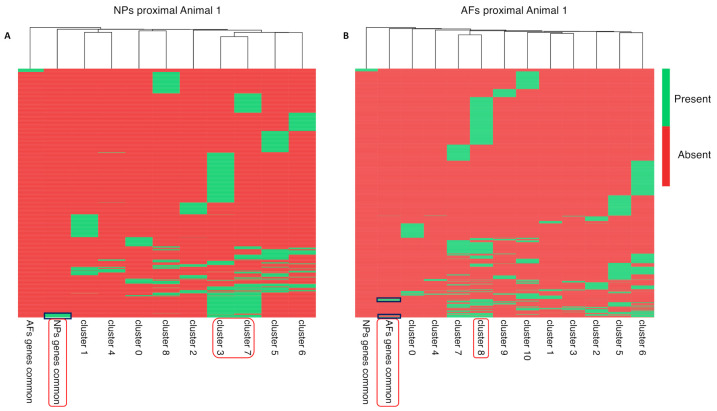
Heat map exclusive genes bulk and scRNAseq. Heat map of exclusive AF and NP genes common between the scRNAseq and bulk RNAseq. These genes are mapped against a single sample. (**A**) The first heat map depicts the cell clusters that express the NP common genes in the sample NPs proximal Animal 1. (**B**) The second one depicts cell clusters that express AF common genes. The dendrogram at the top is a function of the amount of commonly expressed genes.

We then proceeded to identify those same genes in the bulk RNAseq data. We intersected the list of unique specific genes from the scRNAseq data with the bulk transcriptome for AFs and NPs and aligned them to the scRNA cell clusters/populations for NPs and AFs. We performed hierarchical clustering and visualized the results in a heat map (Figure 10). In the NPs, the overlap in structure specific (NP) genes for bulk and single-cells data show the previously identified NP cell Clusters 3 and 7 (Figure 10A). The AF specific genes were added as a control set and did not show any overlap. In the AFs, the bulk and single-cell data overlapped in cell Cluster 8 (Figure 10B). The NP specific genes were added as a control. The dendrogram structure at the top of the heat maps provides a measure of commonly expressed genes among the clusters indicating a potential lineage relationship between clusters. All 27 and 24 structure specific genes identified in the NP and AF respectively were found in the scRNAseq and bulkRNAseq data (Table S5).

### 2.6. Gene Enrichment Analysis

**Figure 11:**
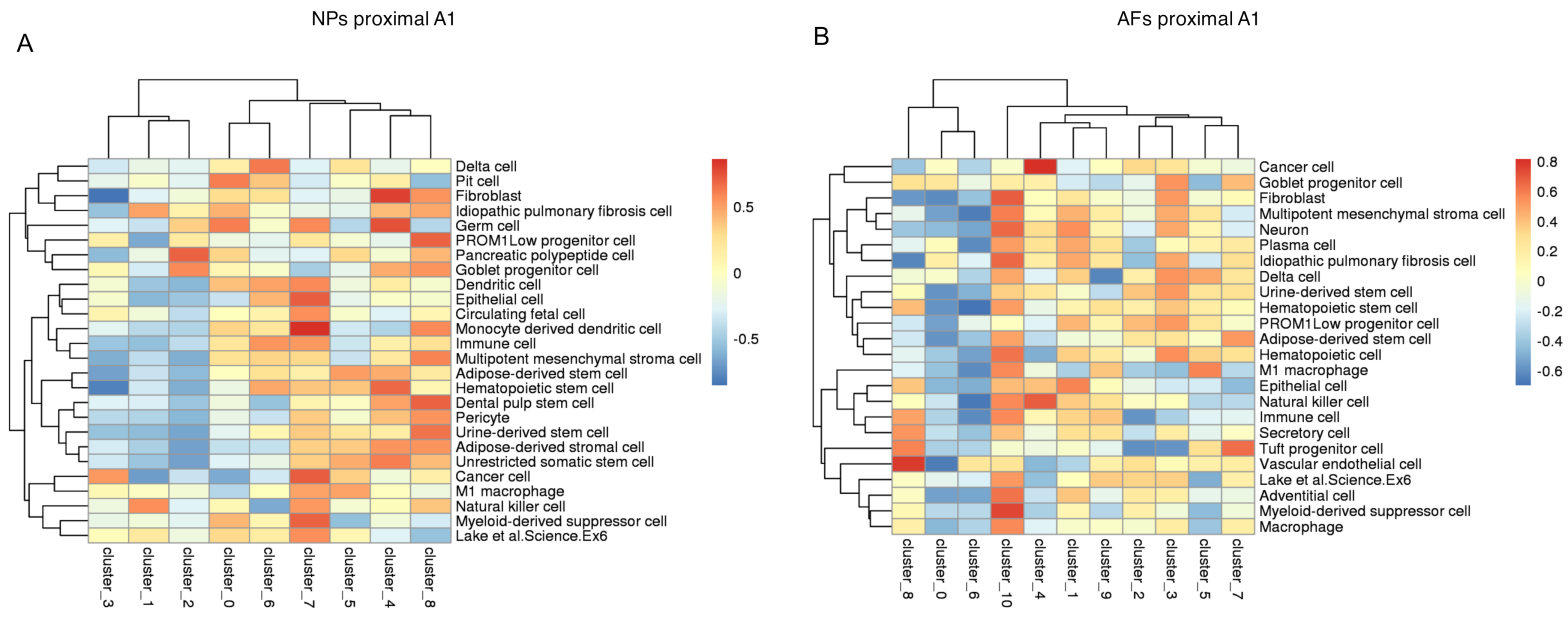
Heat maps enrichment. Heat maps of the enrichment analysis of all the eight scRNAseq samples. The detected genes for each cell cluster have been translated into the human homologous and run through an enrichment analysis. The pathways that could be characteristic of each cluster are highlighted in red, defining a potential cell type profile. (**A**) The first heatmap depicts potential cell type profiles for the NP cell clusters. (**B**) The second heatmap depicts potential cell type profile for the AF cell clusters.

**Figure 12:**
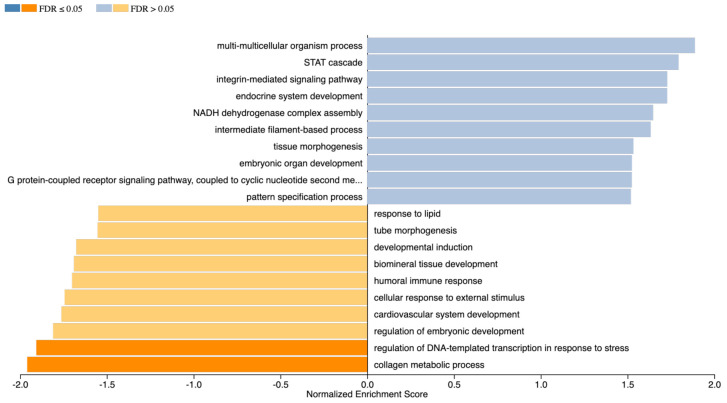
Enrichment analysis bulk RNAseq Enrichment analysis of all the NPs over the AFs bulk RNAseq data. GSEA, GO terms Biological Process. The bar chart shows a significant enrichment of the collagen metabolic process and DNA response to stress in the NPs over the AFs.

Once we identified cell clusters that express specific tissue genes, we aimed at identifying possible cells types for these clusters. The enrichment analysis on a single-cell level allows for the investigation on the potential characteristic of the cell populations in the NPs and AFs tissue. For the gene enrichment analyses, the genes were translated into their human homologous. The subsequent enrichment analysis of each sample hinted at potential cell types for each identified cell cluster (Figure 11). Such hints are especially interesting for the cell clusters that express the structure specific genes, namely Clusters 3 and 7 for the NPs (Figure 11A) and Clusters 7 and 8 for the AFs (Figure 11B). The results for the NP Cluster 3 show the characteristics closest to a cancer cell, yet with otherwise very little common features to the other cells types. These data suggest a cell type that was not present in the repository. Cell Cluster 7 of the NP, however, has the highest similarity to a monocyte derived dendritic cell, followed by cancer cell characteristics. While most suggested cell types are not sensible, the fibroblast characteristics for Clusters 4 and 8 of the NP are plausible. AF Cluster 8 appears to have the highest similarity to vascular endothelial cells. Similar to the NP suggestions, the AF suggested cell types are mostly not convincing. The cell Cluster 10 appears to possess fibroblast-like features, yet its predicted top feature is Myeloid-derived suppressor cell. The consistent presence of stem cell-like cells in the suggestions might hint at the existence of stem and progenitor cells in the clusters. As a last step, we performed an enrichment analysis. The analysis was performed on the single-cell and bulk RNAseq samples. The enrichment allows for the identification of detectable pathways in our samples. We compared all NPs and all AFs using GSEA GO terms Biological process (Figure 12). There is a significant enrichment in the NPs for the collagen metabolic process and regulation of DNA transcription in response to stress.

## 3. Discussion

Single-cell RNAseq enabled the profiling of 92,277 intervertebral individual cell transcriptomes. Our study identified 15 distinct NP cell populations and 12 distinct AF cell populations of the bovine IVDs which supports previous findings on cell population composition of NPs and AFs [34,36,41,43,51,52,53,54,55,56]. Unsupervised cell clustering methods allow the detection of cell types and differentiation states based on the whole transcriptome in a fine-grained manner. These methods have readily and successfully been used [57,58,59,60,61], even if minor imprecisions may occur at defining cross-species identities of specific cell types and functional differences among identified cell types [62,63]. In our single-cell RNAseq analysis, we used the shared nearest neighbor approach for unsupervised cell clustering to identify distinct cell types in the four AFs and four NPs of the bovine IVDs from two animals [64]. Through hierarchical clustering, we identified structure specific genes in the NPs the AFs. These genes were subsequently matched to the earlier identified cell populations. Interestingly, only two distinct NP cell populations and two distinct AF cell populations matched. A literature research on the top expressed genes of each cell population identified two distinct notochordal cell-like populations in the NP and two distinct AFC populations, with one putative fibroblast-like population.

The heterogeneity of the cells contributing to either the NP or AF has been extensively described in the literature. Diverse techniques such as immunohistochemistry, qPCR and Western blot, among others, have been used to define phenotypic markers for these two populations, all of which have their obvious disadvantages, especially in regards to sensitivity issues. The aim of this study was to elucidate at a single-cell resolution the individual NPC and AFC populations along the bovine rostrocaudal model. Coupled with highly sensitive bulk RNAseq analysis of these two tissues, we could identify specific NP and AF genes as well as cell populations, which gives unique insight into the biology of this organ. NPC have been reported to have differentiated along the notochordal lineage, clearly distinguishing them from other cell types in the body [11,12,13]. As a matter of fact, notochordal or notochordal-like cells have been reported in both fetal notochordal immature NP and adult NP. The exact role of these cells remains unclear; however, it is believed that they either act as a regulator or as a progenitor cell for the NP. A broad panel of markers associated with the phenotype of NP notochordal cells [36,38,39,40] was detected mainly concentrated in cell Clusters 3 and 7 from the single-cell NP samples (Figure S8A); these include *CD24*, galectin-3 (*LGALS3*), cytokeratin 8 (*KRT8*), *KRT18, KRT19* and brachyury. Brachyury is a protein that is encoded by the *TBXT* (T-box transcription factor T) and has been described to be necessary for notochordal morphogenesis and patterning. *TBXT* was detected in cell Cluster 7 only, and even some of its binding partners were detected in cell Clusters 3 and 7, such as *SMARCA4, STAT3* and *WNT5B*. We additionally found other notochord associated genes expressed, such as *CDH2* and *FN1*. Galectin-3, however, was predominantly expressed in mainly cell Clusters 8 and 0. Moreover, these cell clusters also expressed genes that have been associated with pluripotent stem cells or progenitor cells such as *KRT15* [65], *CD44* and *CD55*. These two cell clusters are in the same trajectory in the slingshot (Figure S8C) which is a cluster-based lineage reconstruction and pseudotime inference approach indicating their relatedness. These first set of findings, provide a unique insight in the rostrocaudal bovine model for IVDs as we could detect specific cell populations that have a notochord-like phenotype. It is believed that these cell populations decrease as adulthood arrives, as supported by the findings that *CD24* gene expression is reduced in the adult NP [36]. A target of *TBXT* that may be relevant to NPC is the connective tissue growth factor (CTGF/CCN2), which is an ECM protein that is necessary for post-natal NP function [66]. *CTGF* was detected in cell Clusters 1, 2 and 5.

There was an additional cell cluster (Cluster 8) that did not express the notochordal associated genes but instead stem cell markers such as *CD63, CD44* and galectin-3. This cell cluster additionally expressed *WISP2* (*CCN5*), which is a growth factor member of the *CTGF/CCN* family of secreted, ECM-associated proteins [67] that has been shown to be an inhibitor of angiogenesis [68] and predicted to be an active regulator of notochordal gene expression. WISP2 was identified in non-chondrodystrophic dog notochordal cell model (together with CTGF) and proposed to be key for healthy NP homeostasis. Additionally, *NOV*(*CCN3*) was also found in cell Cluster 8. This secreted extracellular protein is key in mesenchymal stem cell differentiation and regulates differentiation, growth and maturation of osteogenic, chondrogenic and hematopoietic progenitor cells. Wnt signaling has been extensively described in the maintenance of notochord progenitor cells identity [69]. The expression of *CCN2* and *CCN5* are upregulated in the early stages of mesenchymal stem cell differentiation by Wnt3A stimulation [70,71]. These results are not surprising since the Wnt/beta catenin signaling pathway has been shown to be required for the development, organization, senescence and degeneration of the NP [72,73].

Thus, cell Cluster 8 indeed indicates a progenitor-stem cell phenotype, as shown in Figure 11. One of the proposed roles for this cell cluster, which needs further interrogation, is their potential role in regulation of notochordal cells. It has been shown that sustained Wnt signaling is required to maintain the notochordal progenitor cells’ notochordal fate [69]. Additionally, cell Clusters 3, 7 and 8 expressed ECM regulators such as *BMP2* and secreted factors (Figure S6), indicating their involvement in NP homeostasis.

Cell Clusters 3 and 7 furthermore expressed *HIGD2A* (HIG1 Hypoxia Inducible Domain Family Member 2A), which is a hypoxia marker, and its expression is induced by the hypoxia-inducible factor-1 (*HIF1*). Cell Cluster 7 additionally had an upregulation of XBP1 (X-box binding protein), a transcription factor and signal transducer involved in HIF1-alpha mediated hypoxia responses in survival [74], thus further supporting that these cell populations play in potentially detecting the environmental condition, ECM regulators and secreting soluble factors to maintain tissue homeostasis as indicated by the matrisome enrichment (Figure S6) [75,76,77].

Since the NP is the largest avascular tissue in the body and NPC have developed to thrive in a unique hypoxic and hyperosmotic environment, we looked further at other hypoxic factors other than *HIGD2A* which also contribute to top cell marker clusters. Cell Cluster 5 of the NP had a higher expression of *HIF1A*, which could potentially indicate that these cells may act as environmental/hypoxic regulators. This is of particular importance since *HIF1A* encodes for the alpha subunit of a transcription factor Hypoxia-inducible factor (HIF-1). HIF-1 functions as a master regulator of cellular and systemic homeostatic response to hypoxia by activating transcription of many genes. Our data show an upregulation in HIF-1A-downstream genes reported to be involved in autophagy (*BNIP3* and *BNIP3L)*, glycolysis (*LDHA* and *PGK1*), cell migration and adhesion (CXCR4), angiogenesis (VEGFA) and lipid storage and endoplasmic reticulum homeostasis (PLIN2). Furthermore we identified *C1QTNF3* and *TXNIP* in cell Clusters 3 and 7 which are in the same trajectory of the slingshot (Figure S8D). *C1QTNF3* has been described as downstream factor of HIF-1alpha which plays an essential role in cartilage development [78]. TXNIP as part of the TXNIP-NLRP3 inflammasome has been related to IVD degeneration [79], yet it is known for its pleiotropic effects as it plays an important role in the energy metabolism [80] and in cell cycle regulation [81]. Semaphorin 4A (*SEMA4A*) was also expressed in cell Cluster 5. Semaphorins are transmembrane proteins that have been shown to not only lead to repulsive axon guidance but also to play key roles in tissue homeostasis and morphogenesis. These results potentially indicate that cell Cluster 5 is one of the NPC populations involved in NP homeostasis under hypoxic conditions and avoiding nerve ingrowth. It is also a contributor to the ECM by expressing regulators, glycoproteins and proteoglycans (Figure S6). This same Cluster 5 expresses T-cell factor 3 (*TCF3*), a member of the Wnt signaling pathway that has been shown to be a modulator in embryonic stem cells and development [82]. In regards to the ECM, in the NP, we observed that collagens were primarily expressed in cell Clusters 1, 4 and 8 (Figure S6), which accounted for almost a third of all the cells analyzed. Additionally, returning to the point about the effects that hypoxia may have in maintaining tissue homeostasis. HIF1-alpha has been shown to control collagen synthesis in chondrocytes by inducing collagen-modifying enzymes such as lysil oxidase (*LOX*) and collagen prolyl hydroxylase (*P4H42*), these genes were expressed in cell Cluster 8 and 4, respectively [83,84,85]. These are key ECM-modifiers that increase crosslinking and stability of the collagen triple helices. Taken together, our data indicate a series of very distinct cell populations that are closely orchestrated living in this unique environment. Our data, whether in the single-cell RNAseq or the bulk RNAseq, show that we could identify most NP specific markers and genes previously reported to be expressed at higher levels in the NP than in the AF [86,87].

From the eight differentially expressed genes in the NP reported in an earlier single-cell experiment from Fernandes et al. 2020, we could detect six genes in our single-cell RNAseq data, namely *COL2A1, KRT8, CD24, COMP, FMOD* and *ACAN*, but not *A2M* and *DSC3*. Discrepancies between their single-cell experiments and ours are most likely due to single-cell RNAseq experimental/technique, number of cells analyzed and sequencing differences, especially in regards to sequencing depth. Furthermore, our bulk RNA experiments could only support the overexpression of KRT8 and CD24 in the NP, indicating the potential limitations in detecting changes in gene expression in very heterogeneous cell populations. Novel NP markers such as *CHRD* [88], *CHRDL2, SCUBE1* [89] and *CLEC3B* were both detected and differentially expressed in both our NP datasets [21]. Some of the novel detected NPC contributing genes are part of important signalling pathways and act as inhibitors of WNT (*FRZB* and *DKK3*) and BMP signaling (*CHRD, CHRDL2*).

The AF provides a wrapping with strong tensile properties and contains the NP. This leads to a unique architecture of the AF as it is composed of a ring of ligament fibers with a tendon/ligament-like structure [90]. Thus, the cells in the AF reported very specific phenotypic signatures that can enable such a defined biomechanical AF function. In contrast to the NP, where only a couple of cell clusters expressing some collagens, most cell clusters in the AF contributed by expressing of a wide variety of collagens (Figure S7). Among those highly expressed collagens, we found *COL1A1, COL1A2, COL9A3, COL18A1* and *COL4A1*, which have been widely reported [20,21].

Small leucine rich proteoglycans (*SLRPs*) such as *BGN, DCN, FMOD, OGN* and *PRELP* were highly expressed in the AF and predominantly found to be expressed in Clusters 1 and 6 (Figure 8B). SLRPs are associated with collagen fibrillogenesis and ECM assembly especially in the AF to form concentric lamellae of oriented collagen [22,23,24].

Another major component of the ECM are the basement membrane proteins and a major noncollagenous constituent of this family are laminins which have been described in both chondrocytes and the IVD. *LAMA3/4/5*, *LAMB1/2/3* and *LAMC1* were top cluster markers for a wide range of cell clusters in the AF.

From the ECM remodeling side, we detected *SPARC* (*SPARC*, also called osteonectin or basement membrane protein 40), which encodes a cysteine-rich acidic matrix-associated protein. This matricellular protein plays a dynamic role in regulating extracellular procollagen processing in the pericellular environment so that it can be efficiently incorporated into the ECM [91,92,93]. SPARC has been proposed to modulate cell-ECM interactions and remodeling of the ECM since it binds collagen Types I–V and VIII, vitronectin and thrombospondin-1 [94,95,96]. Increased methylation of *SPARC* and its promoter as well as decrease in levels of *SPARC* have been reported in the human IVD with aging and in patients suffering with low back pain [97,98]. In the AF, *SPARC* was highly differentially expressed in the AF over the NP and ubiquitously found in the scRNAseq data for the AF, indicating its pivotal role for a healthy AF environment.

Other matricellular genes involved in collagen fibrillogenesis were detected in our AF data and include *PCOLCE*, *PCOLCE2* and *MXRA5* [21]. Due to load bearing properties of the AF and similarities in mechanical functionalities, it was not surprising to find that AFC expressed tendon-related markers such as Tenacin C, tenomodulin and thrombosponins (*THBS1/3/4*) as well as cartilage intermediate layer proteins (*CILP2* and *CILP*), some of which have reported in previous studies [21,99]. Additionally, a wide variety of heat shock proteins (*HSPA5, HSPA6, HSPA8, HSP90AA1, HSP90AB1, HSP90B1, HSPB1, HSPH1* and *HSPA1A*) were mostly detected in Clusters 4 and 5 in the AF, suggesting their role in the stress response to load bearing.

Growth factors such as *CTGF, PDGFB, VEGFC, IGF2 and FGF2/7* were also detected in the AF mostly concentrated in cell Cluster 8. According to the potential cell type profile (Figure 11), the cells in Cluster 8 have a vascular endothelial and progenitor cell phenotype. We found though that it also expresses genes associated with class II major histocompatibility complex (MHC) such as *CD74* and *BoLA-DR-alpha*. This same cluster highly expresses *CXCR4* that is involved in the recruitment of mesenchymal stem cells in bone fractures and *IGF2* that is related to fibroblast proliferation [100,101].

We detected **TIE1** and **TIE2**/**TEK** in the AF specifically in cell Cluster 8. Intriguingly, TIE1 and TEK receptor kinases have been reported to be specifically expressed in endothelial cells during embryonic angiogenesis [102]. Through heterodimerisation, they mediate interactions not only with the ECM but also the surrounding mesenchymal cells, thus suggesting a paracrine regulation [103]. VEGF has also been shown to activate TEK through TIE1 [104]. These findings are significant as up to now TEK/TIE2+ cells have been uniquely described in progenitor NPC across many species, including bovine and human [26].

Surprisingly, cell Cluster 9 in the AF seems not to be a major contributor to the matrisome (Figure S7), although it highly expressed *CD44, CD63* and galectin-3. indicating a stem cell-like phenotype which may play key role in tissue homeostasis. Furthermore, *CALD1, LUM* and *CTSK,* all of which have been described in fibroblasts of the dermis [105], were highly expressed in cell Clusters 9 and 10, as was *PTGDS*, which has been recently described as a papillary fibroblast marker in the human dermis [106]. The potential cell type profile of Cluster 10 is fibroblast-like cells. *TAGLN* (*SM22*) has similarly been described in fibroblasts and dedifferentiated cultured chondrocytes [107,108]. Overall, our data match spatial proteomics findings from Tam V. 2020 in detecting structural specific genes both in the NP and AF, indicating the relevance and integrity of our data [21]. Discrepancies, however, might be due to the disc composition and remodeling of a sample, especially because with RNAseq data we only capture the cellular information and these only represent a small fraction of the disc volume [109].

Taken together, our results show a complex and heterogeneous cell population in both the NP and AF.

Calf tails have been extensively used in intervertebral disc studies as a ready source of these cell populations [41,110,111,112]. The use of this model in our study, however, does entail some limitations specially in regards to the age of the calves as there is a variable window of 6 months. Given the rostrocaudal developmental gradient in calves, an age shift can influence the cellular composition of the IVDs at a given level, thus introducing variance in the differential gene expression analysis of the proximal and distal IVDs [44]. However, we may have compensated for the age-related variance due to the pooling of three adjacent IVDs to obtain enough cells for our experiments. Our scRNAseq data suggest, especially for the AF, a significant developmental gradient along the rostrocaudal axis. Indeed, we showed size fluctuations of 10–20% exist in the larger cell cluster fractions of the NPs and AFs. These fluctuations are expected mainly due to age differences when comparing AFCs and NPCs among different animals. Furthermore, the amounts of fluctuation among animals is larger for the NPCs and along the rostrocaudal axis for the AFs. Given that these cell populations may consist of stem cells, transient amplifying progenitors and terminally differentiated cells, the pattern of these fluctuations may indicate possible temporal differences in the development of the two compartments. This would be consistent with the transcripts expressed by certain populations of these intervertebral disc cells. This notion is further supported by the differential gene expression analysis performed on the so-called bulk RNAseq data from three additional calves. While the NP samples showed no distinct patterns across animals along the rostrocaudal axis, the AF samples showed significant differences along the rostrocaudal axis but not across animals. These differences consist of three differentially expressed transcript clusters being upregulated in the proximal AF in all animals and the other three clusters being upregulated in the distal compartment. Furthermore, hierarchical clustering in the bulk RNAseq showed, as expected, major transcriptome profile differences between the AF and NP, as previously published [20]. Overall, our data suggest that the AF exhibits rostrocaudal developments whereas the NP seems to be rather stable.

Another potential drawback from the IVD cell isolation procedure used in our study is the lack of hypoxic conditions during the extracellular matrix digestion to obtain the single-cell suspension required for the scRNAseq experiments. We are aware that this could have influenced the differentiation state of the cells and as a result altered the cell cluster sizes and numbers [113]. However, IVD cells were immediately processed after digestion to ensure a minimal impact on the transcriptome of the IVD cells themselves as well as the maintenance of the original cell numbers. Multiple studies have shown that culturing IVD cells in monolayers after digestion affect IVD cell phenotype and differentiation [113,114,115,116].

## 4. Materials and Methods

### 4.1. Samples Collection and Processing

Two bovine tails were used for the scRNAseq, and three tails for the bulk RNAseq. The animals were aged from 6 to 12 months. The cow tails were collected from local abattoir within 2 h post mortem, during which the samples were kept at 4 degrees. After disinfection of the tails with Betadine (Mundipharma Medical Company, Basel, Switzerland), the IVDs were dissected and small portions of the Nuclei Pulposi and the Annuli Fibrosi were extracted. For the scRNAseq, the three most proximal NPs were pooled, as well as the three most distal ones, in order to have a minimum of 30,000 cells per sample (Table 1). The same procedure was applied to isolate the most proximal and distal AFs. As it was previously shown that there are gene expression differences noted depending on the segmental level of the coccygeal discs [117], we decided to pool the most distal and the most proximal IVD tissues. For the bulk RNAseq, only the two most proximal and distal NPs and AFs were used (Table 2). The samples were then cut in small pieces and incubated in flasks with 0.19% Pronase for 1 h at 37${}^{\circ }$ on shaker at lowest speed. After the incubations, the cells were then washed with PBS before placing them again in flasks for an overnight incubation with Collagenase II (260 IU/mg) at 37${}^{\circ }$ on shaker at lowest speed. The next morning, the cells were washed with PBS and then filtered through a 100 μm filter to remove potential undigested tissue aggregates and debris. For the scRNAseq samples, the cells were then counted and diluted in PBS + BSA (0.05%) with an approximate concentration of 1000 cells/μL. For the bulk RNAseq samples, the cells were collected in a pellet and shock frozen.

### 4.2. Bulk RNAseq

The RNA was extracted following the RNAeasy Mini Kit Qiagen manual. The quality of the RNA was evalutated using Tapestation (Agilent, Waldbronn, Germany). The libraries were prepared using SENSE mRNAseq Library Prep Kit (Lexogen, Vienna, Austria) and sequenced on the NextSeq500 System (Illumina, San Diego, CA, USA) using a 150 cycle kit, single-end 150 bp, following the manufacturer’s instructions. In total, 25M reads were obtained per sample.

### 4.3. Bulk RNAseq Data Analysis

The quality of Illumina SE RNAseq reads was evaluated using FastQC v.0.11.7 [118]. FastqScreen v.0.11.1 [119], was used to screen for potential sample contaminations (genomic DNA, rRNA, mycoplasma, etc.) using a custom database including UniVec [120], refseq mRNA sequences, selected genome sequences (human, mouse, bacteria, virus, PHIX, lambda and mycoplasma) [121] and SILVA rRNA sequences [122]. Illumina SE reads were preprocessed using Trimmomatic v.0.36 to trim off sequencing adapters and low-quality ends [123]. Trimmed reads were aligned to the reference genome and transcriptome (FASTA and GTF files, respectively, Ensembl, *Bos taurus*, UMD_v3.1, Annotation release 92-2018-0530) with STAR [124] version 2.7.3a with default settings for single end reads. PCR duplicates were marked using Picard v.2.18.0. Differentially expressed genes were identified using R/Bioconductor package edgeR (R version: 3.6.1, edgeR version: 3.28.1) [125], in which the normalization factor was calculated by trimmed mean of M values (TMM) method [126].

### 4.4. Single-Cell RNAseq

**Figure 13:**
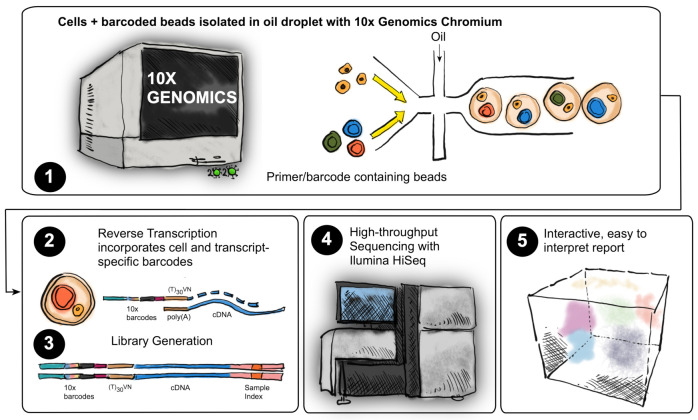
10× single-cell sequencing workflow. Single-cell RNA-seq workflow. (**1**) Isolated single NP or AF cells together with barcoded beads and reverse transcription reagents were isolated in oil droplets using the 10× Genomics Chromium system (**2**) Within each micro reactor, the single cell were lysed, and reverse transcription of polyadenylated mRNA occurs with the incorporation of cell-specific and transcript specific barcodes or UMIs (Unique Molecular Identifier). (**3**) As a result, all cDNAs from a single cell had the same barcode, which enabled the sequencing reads to be clustered together. Next generation sequencing (NGS) libraries from these barcoded cDNAs were prepared. (**4**) NGS was performed in a Novaseq 6000 system. (**5**) Cell Ranger (v3.0.1) was used to align the reads to the reference genome and generate count tables of UMIs for each gene per cell. Subsequent data analysis steps were performed as described in Section 4.5.

The quality and concentration of the single-cell preparations were evaluated using an hemocytometer in a Leica DM IL LED microscope and adjusted to 1000 cells/μL. 10,000 cells per sample were loaded in to the 10× Chromium controller (Pleasanton, CA, US) and library preparation was performed according to the manufacturer’s indications (single-cell 3’ v3 protocol). The resulting libraries were sequenced in an Illumina NovaSeq sequencer according to 10× Genomics recommendations (paired-end reads, R1 = 28, i7 = 8, R2 = 91) to a depth of around 50,000 reads per cell (Figure 13).

### 4.5. Single-Cell RNAseq Analysis

The fastq files were aligned to the *Bos taurus* reference sequence (UMD-v3.1 Release-92) taken from Ensembl. After the alignment, each observed barcode, UMI, gene combination was recorded as a UMI count matrix that was then filtered to remove low RNA content cells or empty droplets. All these steps were performed using the CellRanger software (v3.0.1). Starting from this matrix, we used the R package Seurat (version 2.3.4) [127] to perform the following downstream analyses per sample: genes and cells filtering, normalization, feature selection, scaling, dimensionality reduction, clustering and differential expression. We started by filtering out genes that did not obtain at least 1 UMI count in fewer than five cells, and discarded cells for which fewer than 400 genes or more than 4200 genes were detected and also those that had a mitochondrial genome transcript ratio greater than 0.25. After this, the data were normalized using a global-scaling normalization method that normalizes the feature expression measurements for each cell by the total expression, multiplies this by a scale factor (10,000 by default) and log-transforms the result. We next calculated a subset of 2000 features that exhibited high cell-to-cell variation in the dataset. Using as input these variable features, we performed PCA on the scaled data. Since Seurat clusters cells based on their PCA scores, we used a heuristic method called ‘Elbow plot’ to determine how many principal components (PCs) we needed to capture the majority of the signal. In this way, the cells were clustered using an unsupervised graph-based clustering approach using the first 12 PCs and a resolution value that ranged from 0.3 to 0.5. Clusters were visualized using t-distributed Stochastic Neighbor Embedding of the principal components (spectral t-SNE) [128] as implemented in Seurat. We found positive markers that defined clusters compared to all other cells via differential expression. The test we used was the Wilcoxon Rank Sum test, which assesses separation between the expression distributions of different clusters. Genes with, on average, at least 0.25-fold difference (log-scale) between the cells in the tested cluster and the rest of the cells and an adjusted *p*-value < 0.05 were declared as significant. Cell-cycle phases were predicted using a function included in the scran R package [129] that scores each cell based on expression of canonical marker genes for S and G2/M phases. Since scran only includes markers for human and mouse, we performed this analysis using the human homolog genes taken from Ensembl.

### 4.6. Pathways Analyses

To identify which kind of cell populations were present in the samples, we performed three types of enrichment analyses: singular enrichment analysis (SEA), gene set enrichment analysis (GSEA) and gene set variant analysis (GSVA). GSEA was also performed for the bulk RNAseq data to detect pathways enriched in either the NP or the AF structures. The most traditional enrichment approach, SEA, iteratively tests annotation terms one at a time against a list of interesting genes for enrichment. In this case, we used Gene Ontology 1, KEGG 2 and Wiki pathways 3 as annotation terms and we used the marker genes found in every cluster as our lists of interesting genes. SEA calculates an enrichment p-value by comparing the observed frequency of an annotation term with the frequency expected by chance; individual terms beyond some cut-off (e.g., *p*-value ≤ 0.05) were deemed enriched. GSEA method is similar to SEA, but it considers all genes during analysis, not just those deemed as interesting or significant by some metric or threshold. Alternatively, we performed a biological theme comparison among gene clusters with statistical analysis of GO and KEGG pathways. Finally, we used the enrichment method GSVA to find associations between clusters and cell types. GSVA calculates sample-wise gene set enrichment scores as a function of genes inside and outside the gene set, analogously to a competitive gene set test. GSVA is similar to GSEA, but it estimates variation of gene set enrichment over the samples independently of any class label. This time, instead of using GO terms or KEGG pathways as gene sets, we used cell types and gene markers identified in different tissues in human taken from the CellMarker database 4. *Bos taurus* gene symbols were converted to their homolog gene symbols in *Homo sapiens*.

1. http://geneontology.org (accessed on 29 April 2019)
2. https://www.genome.jp/kegg (accessed on 29 April 2019)
3. https://www.wikipathways.org (accessed on 29 April 2019)
4. http://biocc.hrbmu.edu.cn/CellMarker (accessed on 29 April 2019)

### 4.7. Multiple Samples Integration

To find inter- and intra-individual differences between animals, we used a method implemented in Seurat v 3.0.1 [64] that aims to identify shared cell states that are present across different datasets. We initiated this process through dimensional reduction using canonical correlation analysis (CCA), aiming to place datasets in a shared low-dimensional space. Following dimensional reduction, we identified the K-nearest neighbors (KNNs) for each cell within its paired dataset, based on the L2-normalized CCV. Finally, we identified mutual nearest neighbors (MNN; pairs of cells, with one from each dataset, that are contained within each other’s neighborhoods). We refer to these pairwise correspondences as “anchors”. Filtering and scoring anchors steps were performed to mitigate the effects of any incorrectly identified pairs of cells.

## 5. Conclusions

By integrating the scRNAseq data with the bulk RNAseq data to stabilize the clustering results of our study [130,131], we identified 27 NPC structure/tissue specific genes and 24 AFC structure/tissue specific genes. Developmental changes were detected in the AF along the rostrocaudal axis. From our single-cell RNAseq results, we could deconvolute the heterogeneous cell populations in both the NP and the AF. In the NP, we detected a notochordal-like cell cluster and a progenitor stem cell cluster. In the AF, we also detected a stem cell-like cell cluster, a cluster with a predominantly fibroblast-like phenotype and an endothelial progenitor cell like cluster.

## Figures and Tables

**Table 1:** Samples scRNAseq. List of samples used for single-cell sequencing.

Sample N${}^{\circ }$	Description	Source
1	3 NPs proximal	Animal 1
2	3 AFs proximal	Animal 1
3	3 NPs distal	Animal 1
4	3 AFs distal	Animal 1
5	3 NPs proximal	Animal 2
6	3 AFs proximal	Animal 2
7	3 NPs distal	Animal 2
8	3 AFs distal	Animal 2

**Table 2:** Samples bulk sequencing. List of samples used for bulk RNA sequencing.

Sample N${}^{\circ }$	Description	Source
1	2 NPs proximal	Animal 3
2	2 AFs proximal	Animal 3
3	2 NPs distal	Animal 3
4	2 AFs distal	Animal 3
5	2 NPs proximal	Animal 4
6	2 AFs proximal	Animal 4
7	2 NPs distal	Animal 4
8	2 AFs distal	Animal 4
9	2 NPs proximal	Animal 5
10	2 AFs proximal	Animal 5
11	2 NPs distal	Animal 5
12	2 AFs distal	Animal 5

## Data Availability

https://www.ebi.ac.uk/ena/browser/view/PRJEB44744 (accessed on 19 March 2021).

## Supplementary Materials

The following are available at https://www.mdpi.com/article/10.3390/ijms22094917/s1.

## Abbreviations

The following abbreviations are used in this manuscript:

AF	Annulus Fibrosus
AFC	Annulus Fibrosus Cell
CCA	Canonical Correlation Analysis
CEP	Cartilage Endplate
GSEA	Gene Set Enrichment analysis
GSVA	Gene Set Variant Analysis
IVD	Intervertebral disc
KNN	K-nearest neighbors
MNN	Mutual Nearest Neighbors
NP	Nucleus Pulposus
NPC	Nucleus Pulposus Cell
PCA	Principal Component Analysis
RISH	Hybridization on paraffin sections
SEA	Singular Enrichment Analysis
t-SNE	t-stochastic neighbor embedding
UMI	Unique Molecular Identifier
