# Supplementary material for: The Cellular Composition of Bovine Coccygeal Intervertebral Discs: A Comprehensive Single-Cell RNAseq Analysis
Source: Int J Mol Sci. 2021 May 6;22(9):4917. doi: 10.3390/ijms22094917 (PMC8124861; doi:10.3390/ijms22094917)
Supplement: Supplementary file 1 [file ijms-22-04917-s001.zip › ijms-1170765-supplementary.pdf]

Supplementary Materials:

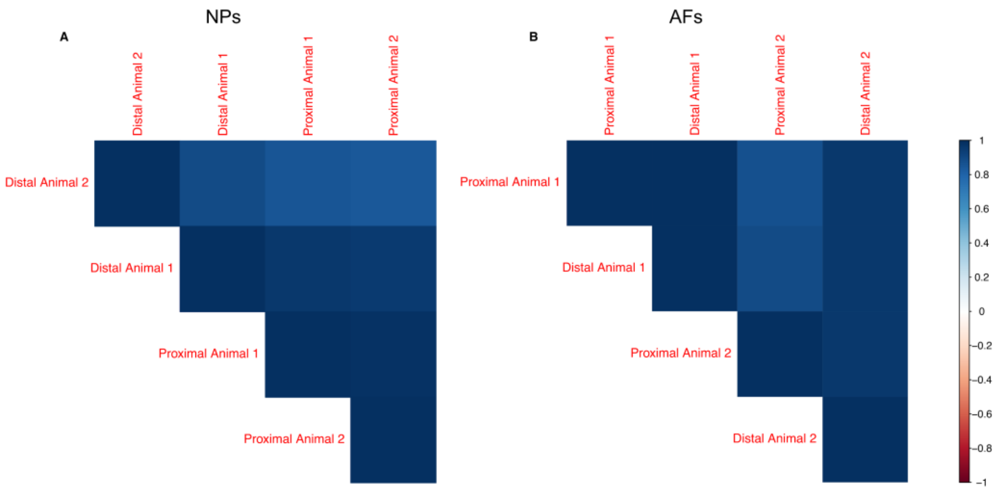

Figure S1. Correlation matrix NPs and AFs for scRNAseq.The matrix shows how correlated the NPs samples and the AFs samples are within the group. The darker is the blue, the higher is the correlation. (A) depicts the correlation between transcriptomes of two animals at different levels in the nucleus pulposus. (B) depicts the correlation between transcriptomes of two animals at different levels in the annulus fibrosus.

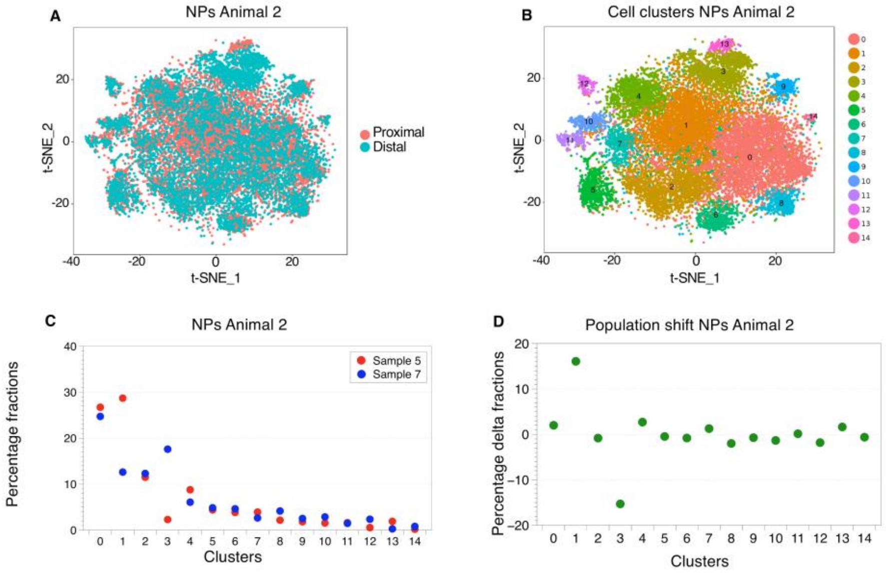

Figure S2. Cell cluster distributions between proximal and distal NPs Animal 2. The first t-SNE plot (**A**) provides a visual measure for similarity for the proximal and distal NPs. The graphical overlap stands for the amount of similarity in gene expression profiles of the distal and proximal NPs. The second t-SNE plot (**B**) shows the identified cell clusters. (**C**) The population sized within a cell cluster. The first bar plot (**C**) is a representation of the percentage of the fractions of cells per cluster, normalized to the total amount of cells in each corresponding sample (see Table S3 for the original data). The population shift observed between the proximal and the distal samples is described in terms of number of cells that compose a cluster in each sample. (**D**) The difference in percentage between the cell cluster sizes of the proximal and distal NPs, indicating the amount of correlation between the samples.

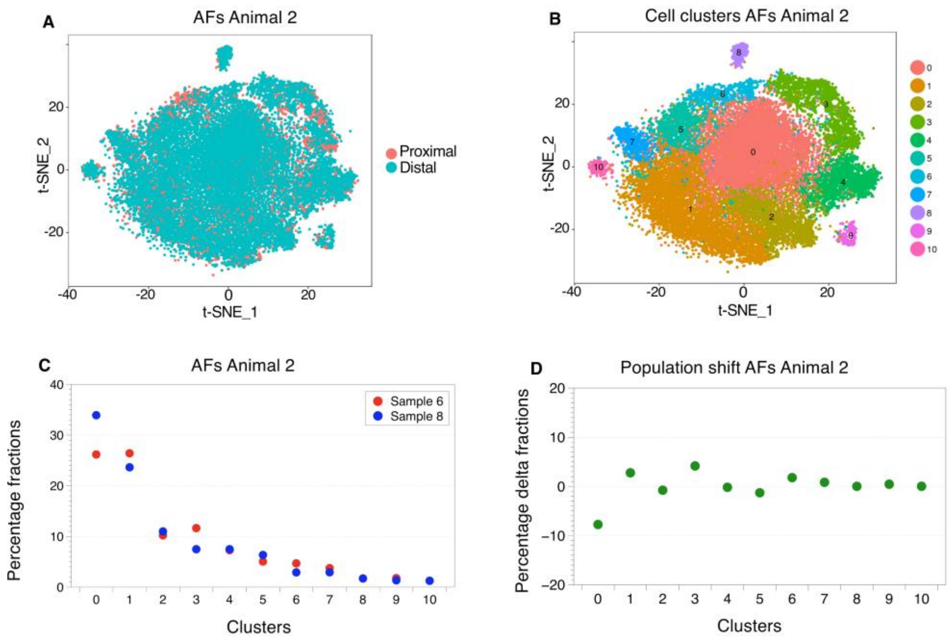

Figure S3. Cell cluster distributions between proximal and distal AFs Animal 2. The first t-SNE plot (**A**) provides a visual measure for similarity for the proximal and distal AFs. The graphical overlap stands for the amount of similarity in gene expression profiles of the distal and proximal AFs. The second t-SNE plot (**B**) shows the identified cell clusters. (**C**) The population sized within a cell cluster. The first bar plot (**C**) is a representation of the percentage of the fractions of cells per cluster, normalized to the total amount of cells in each corresponding sample (see Table S4 for the original data). The population shift observed between the proximal and the distal samples is described in terms of number of cells that compose a cluster in each sample. (**D**) The difference in percentage between the cell cluster sizes of the proximal and distal NPs, indicating the amount of correlation between the samples.

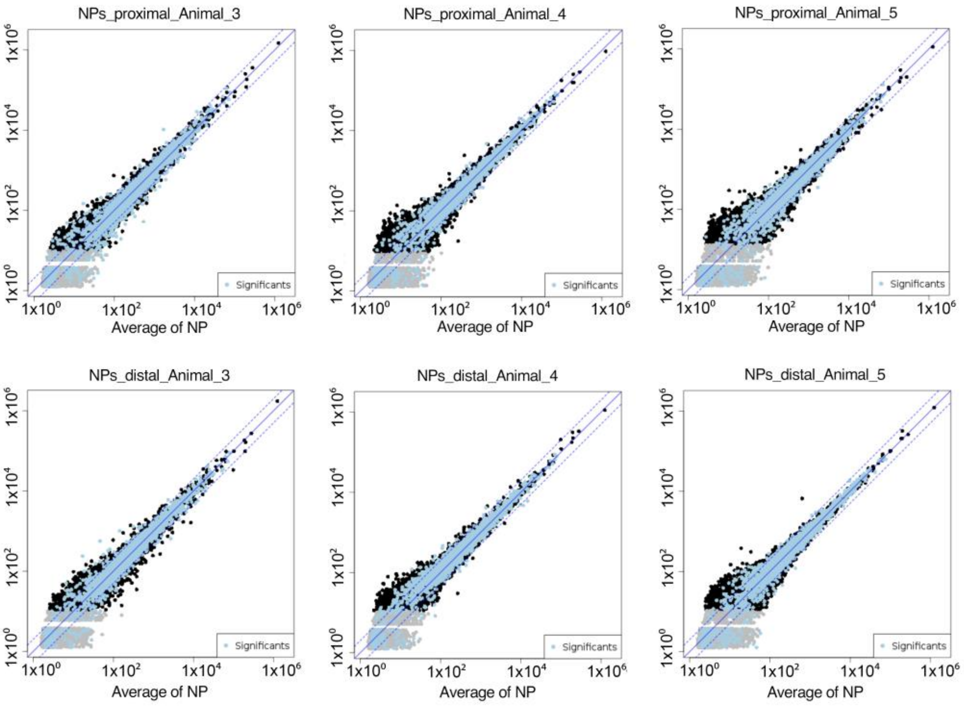

Figure S4. Linear correlation among the NPs samples in the bulk RNAseq. In the matrix, each samples is correlated with the average of the group.

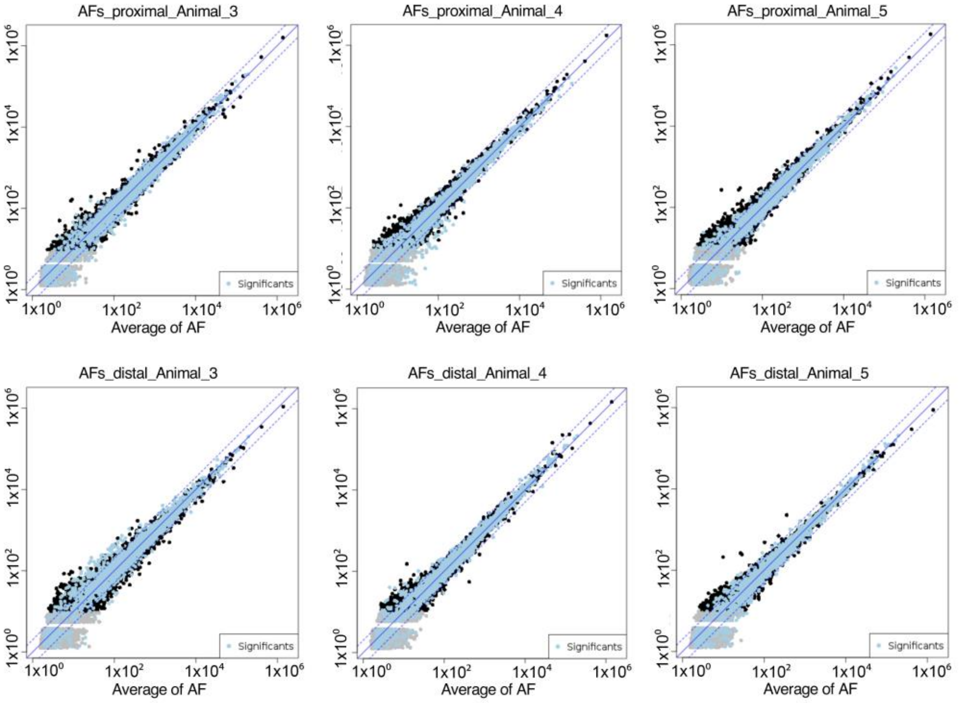

Figure S5. Linear correlation among the AFs samples in the bulk RNAseq. In the matrix, each samples is correlated with the average of the group.

Table S1. Cells per cluster and samples fractions NPs in Animal 1. Sample 1 represents the proximal NPs in Animal 1 and Sample 3 represents the distal NPs in Animal 1.

**Number of cells in each cluster**					
**Cluster**	**# of cells**	**Sample1**	**Sample3**	**Sample1 fraction**	**Sample3 fraction**
0	5634	2084	3550	0.2141829	0.2883132
1	5532	2701	2831	0.2775951	0.2299196
2	2874	1086	1788	0.1116136	0.1452124
3	2275	1050	1225	0.1079137	0.0994883
4	1078	498	580	0.0511819	0.0471047
5	903	352	551	0.0361768	0.0447495
6	843	559	284	0.0574512	0.0230651
7	688	321	367	0.0329908	0.0298059
8	550	242	308	0.0248715	0.0250142
9	461	180	281	0.0184995	0.0228214
10	340	174	166	0.0178828	0.0134817
11	273	170	103	0.0174717	0.0083651
12	236	178	58	0.0182939	0.0047105
13	207	64	143	0.0065776	0.0116137
14	149	71	78	0.0072970	0.0063348
**Total**	22043	9730	12313	1.0000000	1.0000000

Table S2. Cells per cluster and samples fractions AFs in Animal 1. Sample 2 represents the proximal AFs in Animal 1 and Sample 4 represents the distal AFs in Animal 1.

**Number of cells in each cluster**					
**Cluster**	**# of cells**	**Sample2**	**Sample4**	**Sample2 fraction**	**Sample4 fraction**
0	8930	3624	5306	0.3739938	0.3695243
1	4869	1916	2953	0.1977296	0.2056550
2	2564	1095	1469	0.1130031	0.1023052
3	2137	781	1356	0.0805986	0.0944355
4	1912	810	1102	0.0835913	0.0767463
5	872	405	467	0.0417957	0.0325232
6	714	281	433	0.0289990	0.0301553
7	635	133	502	0.0137255	0.0349607
8	473	204	269	0.0210526	0.0187339
9	372	201	171	0.0207430	0.0119089
10	348	103	245	0.0106295	0.0170625
11	223	137	86	0.0141383	0.0059893
**Total**	24049	9690	14359	1.0000000	1.0000000

Table S3. Cells per cluster and samples fractions NPs Animal 2. Sample 5 represents the proximal NPs in Animal 2 and Sample 7 represents the distal NPs in Animal 2.

**Number of cells in each cluster**					
**Cluster**	**# of cells**	**Sample5**	**Sample7**	**Sample5 fraction**	**Sample7 fraction**
0	5288	3188	2100	0.2673599	0.2473207
1	4495	3423	1072	0.2870681	0.1262513
2	2418	1372	1046	0.1150621	0.1231893
3	1770	275	1495	0.0230627	0.1760688
4	1563	1047	516	0.0878061	0.0607702
5	938	525	413	0.0440288	0.0486397
6	852	458	394	0.0384099	0.0464021
7	693	468	225	0.0392486	0.0264986
8	611	258	353	0.0216370	0.0415734
9	431	216	215	0.0181147	0.0253209
10	428	184	244	0.0154311	0.0287363
11	318	193	125	0.0161858	0.0147215
12	268	67	201	0.0056189	0.0236721
13	250	227	23	0.0190372	0.0027088
14	92	23	69	0.0019289	0.0081263
**Total**	20415	11924	8491	1.0000000	1.0000000

Table S4. Cells per cluster and samples fractions AFs Animal 2. Sample 6 represents the proximal AFs in Animal 2 and Sample 8 represents the distal AFs in Animal 2.

**Number of cells in each cluster**					
**Cluster**	**# of cells**	**Sample6**	**Sample8**	**Sample6 fraction**	**Sample8 fraction**
0	8213	2939	5274	0.2618030	0.3392949
1	6641	2965	3676	0.2641190	0.2364900
2	2856	1147	1709	0.1021735	0.1099460
3	2470	1307	1163	0.1164262	0.0748199
4	1982	819	1163	0.0729556	0.0748199
5	1554	567	987	0.0505077	0.0634972
6	978	526	452	0.0468555	0.0290787
7	875	421	454	0.0375022	0.0292074
8	458	193	265	0.0171922	0.0170484
9	408	200	208	0.0178158	0.0133814
10	335	142	193	0.0126492	0.0124164
**Total**	26770	11226	15544	1.0000000	1.0000000

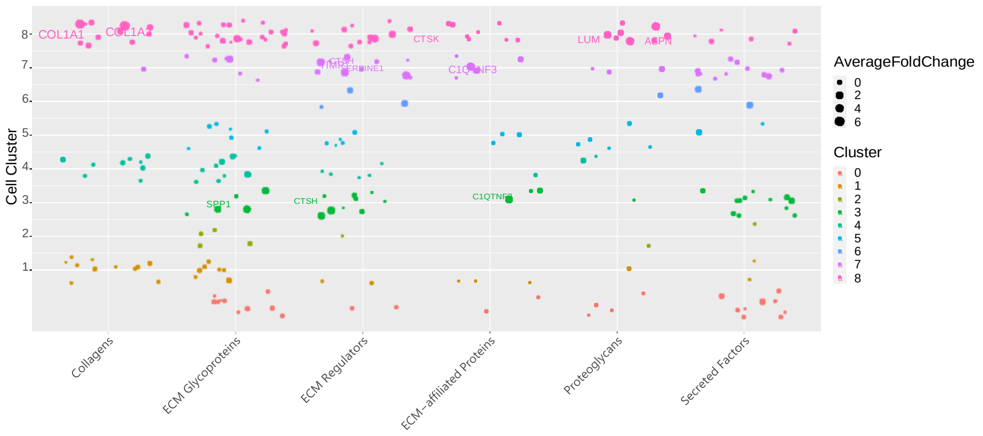

Figure S6. Matrisome Enrichment per cell cluster of the proximal NP of Animal 1. The *x*-axis of the plot shows the category of the Matrisome. The y-axis represents the cell cluster and the dot size represents the fold change of the gene expression.

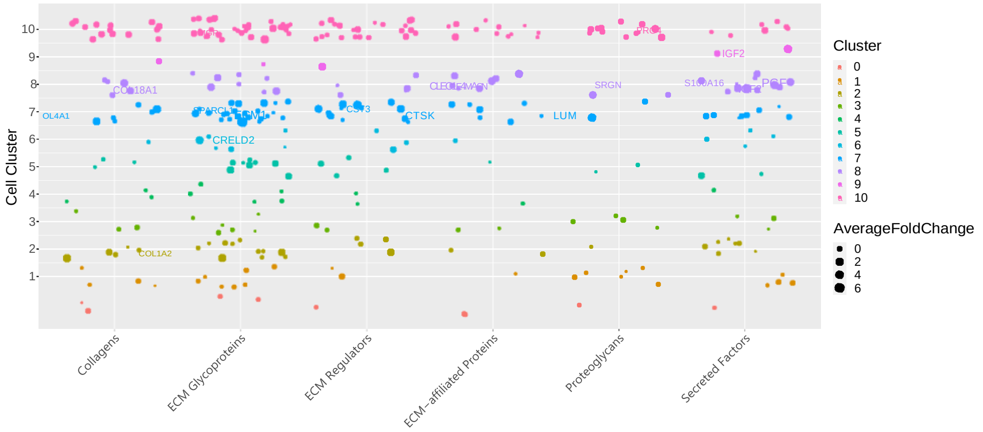

Figure S7. Matrisome Enrichment per cell cluster of the proximal AF of Animal 1. The *x*-axis ofthe plot shows the category of the Matrisome. The *y*-axis represents the cell cluster and the dot size represents the fold change of the gene expression.

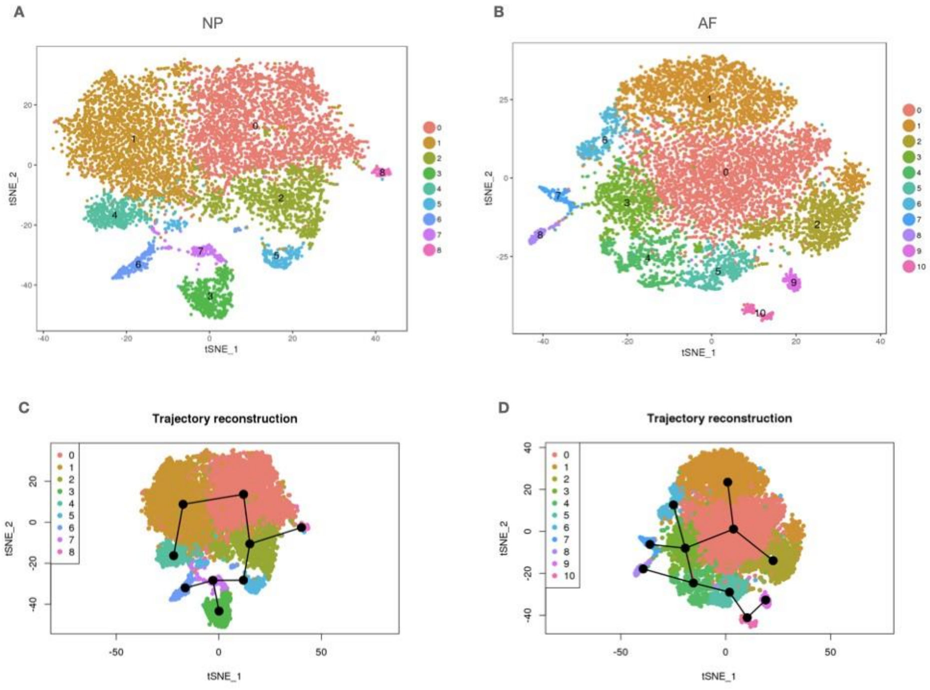

Figure S8. Single Sample Animal 1 NP and AF cell clusters and lineage projection. The figure depicts tSNE and trajectory plots of the NPs and AFs of Animal 1: (**A**) the tSNE plot and the cell clusters identified in the NP; (**B**) the tSNE plot and the cell clusters identified in the AF; (**C**) the reconstructed lineage trajectory of the NP cell clusters; and (**D**) the reconstructed lineage trajectory of the AF cell clusters.

Table S5. Common markers between scRNAseq and bulkRNAseq. The genes that are present in the list of typical genes for the NPCs or AFCs in the scRNAseq and bulkRNAseq data. Based on a gene comparison of the results depicted in Figures 9 and 11 only a limited number of genes matches.

Proximal NPs Animal1 Bulk and scRNAseq	Proximal AFs Animal1 Bulk and scRNAseq
CD96	PTPPR
FLT1	BMP4
PTGR1	ADGRL2
BFAR	TM4SF18
DUSP26	PTPRB
ATG4A	ROBO4
F2RL2	PLVAP
PAK1	CALCRL
PCDH7	LGALS9
FZD2	RSAD2
SOSTDC1	ADGRF5
KRT18	EMCN
CHST4	ADGRL4
ADGRD1	CLEC14A
KIAA1217	PECAM1
AP3B2	SPRY1
TMEM163	CXCL16
KYAT3	RNASE4
PKP2	FGF7
C3orf49	LHFPL2
KRT19	CRABP2
CDH2	TFPI2
DSP	SERPINF1
DDR1	FBLN1
PDE1A	
KRT8	
ATP6V1G3	
